# Effect of Chemical Structure and Salt Concentration on the Crystallization and Ionic Conductivity of Aliphatic Polyethers

**DOI:** 10.3390/polym11030452

**Published:** 2019-03-09

**Authors:** Jorge L. Olmedo-Martínez, Leire Meabe, Andere Basterretxea, David Mecerreyes, Alejandro J. Müller

**Affiliations:** 1 POLYMAT and Polymer Science and Technology Department, Faculty of Chemistry, University of the Basque Country UPV/EHU, Paseo Manuel de Lardizabal 3, 20018 Donostia-San Sebastián, Spain; jorge.olmedo.martinez@gmail.com (J.L.O.-M.); leire.meabe@polymat.eu (L.M.); andere.basterrechea@ehu.eus (A.B.); david.mecerreyes@ehu.eus (D.M.); 2 IKERBASQUE, Basque Foundation for Science, 48011 Bilbao, Spain

**Keywords:** polyethers, crystallization, ionic conductivity, Flory–Huggins theory

## Abstract

Poly(ethylene oxide) (PEO) is the most widely used polymer in the field of solid polymer electrolytes for batteries. It is well known that the crystallinity of polymer electrolytes strongly affects the ionic conductivity and its electrochemical performance. Nowadays, alternatives to PEO are actively researched in the battery community, showing higher ionic conductivity, electrochemical window, or working temperature range. In this work, we investigated polymer electrolytes based on aliphatic polyethers with a number of methylene units ranging from 2 to 12. Thus, the effect of the lithium bis(trifluoromethanesulfone) imide (LiTFSI) concentration on the crystallization behavior of the new aliphatic polyethers and their ionic conductivity was investigated. In all the cases, the degree of crystallinity and the overall crystallization rate of the polymers decreased drastically with 30 wt % LiTFSI addition. The salt acted as a low molecular diluent to the polyethers according to the expectation of the Flory–Huggins theory for polymer–diluent mixtures. By fitting our results to this theory, the value of the interaction energy density (B) between the polyether and the LiTFSI was calculated, and we show that the value of B must be small to obtain high ionic conductivity electrolytes.

## 1. Introduction

Dry solid polymer electrolytes (SPEs) have attracted great attention as safe alternatives to liquid electrolytes in different energy storage technologies, such as lithium batteries for electric vehicles [1,2,3]. SPEs are formed by complexing an ionic salt within a polymer matrix. It is generally accepted that the ionic conductivity occurs in the amorphous part of the polymers, and the ion dynamics are governed by the segmental motion of the amorphous phases in polymers [4]. Several polymers and salts have been evaluated as SPEs, such as poly(ethylene oxide) (PEO), poly(vinyl alcohol) (PVA), poly(methyl methacrylate) (PMMA), poly(*ε*-caprolactone) (PCL), polycarbonates (PC), chitosan (CS), poly(vinylpyrrolidone) (PVP), poly(vinyl chloride) (PVC), poly(vinylidene fluoride) (PVDF), and poly(ionic liquid)s [5,6], among other polymers. Different lithium salts have been employed, such as LiClO_4_, LiBF_4_, LiPF_6_, LiFSI [7], and lithium bis(trifluoromethanesulfone) imide (LiTFSI). In particular, LiTFSI has been widely employed as its low lattice energy favors salt dissolution and dissociation, leading to enhanced ionic conductivity [8].

Among all the polymer matrices, PEO is the most studied polymer electrolyte in lithium batteries. Due to its polarity, lithium salts can be easily dissolved in PEO, a fact that promotes ion mobility [9,10,11]. The PEO/LiTFSI system has been widely studied because of the high dissociation and plasticizing abilities of LiTFSI that leads to better ionic conductivities, as compared to other salts. However, the low ionic conductivity obtained at low temperatures, the low lithium transference number, and the resulting high interfacial resistance are common problems for this electrolyte to be applied in lithium batteries. In these polymer electrolytes, the salt plays two roles; the introduction of ionic charge carriers and the suppression of crystallization of the PEO. Thus, Marzantowicz et al. studied the crystallization of PEO/LiTFSI by polarized optical microscopy and ionic conductivity simultaneously, and they found that the decrease of conductivity during crystallization is related to the reduction of amorphous conductivity pathways by growing spherulites [12]. An in-depth study of crystallization behavior of PEO helps in the understanding of the electrochemical properties of SPEs [13]. Generally, crystallization limits ionic conductivity. Consequently, different strategies have been developed to limit the crystallization of PEO and improve the ionic conductivity in SPEs. Some of the strategies employed in this direction are synthesizing block and random copolymers [14,15], cross-linked polymer electrolytes [16], or adding nano-particles [17].

Furthermore, the crystallization kinetics of polymer electrolytes has a direct effect on the structure and properties of the SPEs. Sim et al. studied the effect of molecular mass of PEO and LiClO_4_ content on the isothermal crystallization of PEO [18]. Their study reports a very large decrease in the isothermal crystallization rate with salt addition. In addition, they observed that the effect is more pronounced as the molecular weight of PEO increased. Additionally, Zhang et al. investigated the crystallization behavior of PCL/LiClO_4_, and the results indicated that Li salts affected the crystallization behavior of PCL without changing its crystalline structure [19].

Very recently, we have reported a new versatile synthetic pathway to a variety of aliphatic polyethers by organocatalyzed bulk self-condensation of aliphatic diols [20]. This prompted us to investigate the effect of the chemical structure of the aliphatic polyethers and the salt concentration in the crystallinity of the polymer electrolytes. In this work, we prepared solid polymer electrolytes (SPEs) composed of aliphatic polyethers, with different methylene numbers in their repeating units (between 2 and 12) and several LiTFSI concentrations (10, 30, 50, and 80 wt %). The objective was to study the effect of the chemical structure of the different polyethers and LiTFSI salt concentrations on the crystallization kinetics and ionic conductivity of polyethers/LiTFSI SPEs.

## 2. Materials and Methods 

### 2.1. Materials

Poly(ethylene oxide) (PEO) (M_W_ 2000 g mol^−1^) and polytetrahydrofuran P(THF) (M_w_ 2000 g mol^−1^) were purchased from Sigma Aldrich (Madrid, Spain). Chloroform (99%) was supplied by Scharlau (Barcelona, Spain) and acetone (99.5 %) by Acros Organics (Madrid, Spain). Finally, the lithium bis(trifluoromethane) sulfonimide (LiTFSI) (99.9%) salt was purchased from Solvionic (Toulouse, France).

### 2.2. Synthesis of the Linear Polyethers

**Figure 1:**
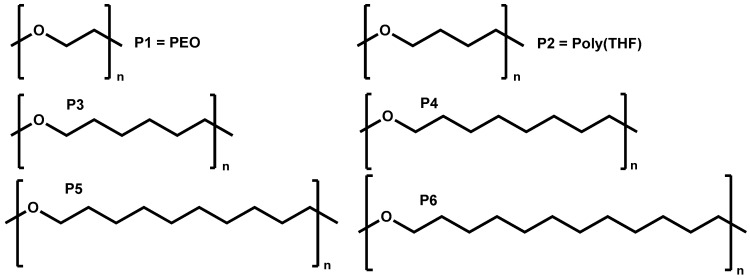
Chemical structure of the different polyethers employed in this work.

The aliphatic polyethers were synthesized following the methodology described by Basterretxea et al. [20]. Direct polycondensation of diols containing 6, 8, 10, and 12 methylene units was performed in solvent free conditions. The reaction was catalyzed by protic ionic liquids previously prepared by mixing methanesulfonic acid (MSA) and 1,5,7-triazabicyclo[4.4.0]dec-5-ene (TBD) in a 3:1 molar ratio. The polymers were named using the following nomenclature: PEO = P1, Poly(THF) = P2, and the synthesized polymers with different numbers of methylene groups in their repeat units are poly(oxyhexamethylene) (P3) > poly(oxyoctomethylene) (P4) > poly(oxydecamethylene) (P5) > poly(oxydodecamethylene) (P6), as shown in Figure 1. 

### 2.3. Elaboration of SPE Solid Polymer Electrolytes

SPEs were prepared by the solvent casting method. Polyethers and lithium salt were dissolved in a chloroform/acetone (90/10 *v*/*v*%) mixture. The solutions were directly casted onto a silicon mold. First, the membranes were dried at ambient conditions and, later, the total evaporation of the solvents was completed applying high vacuum at 90 °C during 24 h. The SPEs were transferred into a nitrogen filled glovebox to assemble the cell. In the first part, SPEs containing 30 wt % of LiTFSI were prepared (0.15 g of polymer and 0.064 g of salt in 3 mL of chloroform). Later, polymers P3 and P5 were selected to prepare SPEs with different concentrations of LiTFSI: 10, 30, 50, 80, and 90 wt % LiTFSI.

## 3. Characterization Methods

The electrolytes were characterized by differential scanning calorimetry (DSC). The experiments were performed in a Perkin Elmer 8500 DSC equipped with an Intracooler III and calibrated with indium and tin standards. For the non-isothermal scans, the heating rate was 20 °C min^−1^ in a range of −60 to 150 °C. Samples between 3 and 5 mg were used. Measurements were performed by placing the samples in sealed aluminum pans. The samples were first heated with a scan rate of 20 °C min^−1^, from 25 to 150 °C and kept for 3 min at 150 °C to erase thermal history, then cooling and subsequent heating scans were recorded at 20 °C min^−1^.

Ionic conductivities were measured by electrochemical impedance spectroscopy (EIS) in an Autolab 302N potentiostat galvanostat (Metrohm AG, Herisau, Switzerland) with the temperature controlled by a Microcell HC station. The SPE was sandwiched between two stainless steel electrodes (surface area = 0.5 cm^2^). The plots were obtained applying a 10 mV perturbation to open circuit potential in the frequency range of 100 kHz to 1 Hz.

Prior to isothermal crystallization analysis, the isothermal *T_c_* range employed for each electrolyte was determined by the procedure recommended by Lorenzo et al. [21]. This procedure was employed in order to avoid the crystallization during the cooling step. Once the starting minimum *T_c_* was determined, the samples were subjected to the following successive stages: (i) heating from 25 to 100 °C at 20 °C min^−1^; (ii) isothermal holding at 100 °C during 3 minutes; (iii) cooling to the selected *T_c_* at 60 °C min^−1^; (iv) isothermal holding at *T_c_* until the crystallization process was saturated, and; (v) heating from the selected *T_c_* to 100 °C at 20 °C min^−1^ in order to register the melting behavior after the isothermal measurement.

## 4. Results and Discussion

### 4.1. Non-Isothermal Crystallization of Aliphatic Polyethers in the Presence of LiTFSI

As mentioned before, poly(ethylene oxide) (PEO) is widely explored in the area of SPEs for lithium batteries [7]. Adequate coordination between different salts and PEO has been investigated for many years [22,23]. The crystallinity of PEO plays an important role, as it has been demonstrated that it can hinder the ionic conductivity. Nevertheless, there are no reports on the ionic conductivity and the effect of crystallinity on other aliphatic polyethers with higher amounts of methylene units. For this reason, we first examined the thermal properties of pure polyethers and their mixtures with 30 wt % LiTFSI by DSC. In total, six different polyethers have been compared: commercially available PEO (P1) with two methylene units, poly(tetrahydrofurane) PTHF (P2) with four methylene units, and the synthesized aliphatic polyethers with 6, 8, 10, and 12 methylene units, respectively (labeled P3, P4, P5, P6). All the studied polymers have a similar number average molar mass of around 2000 g mol^−1^.

**Figure 2:**
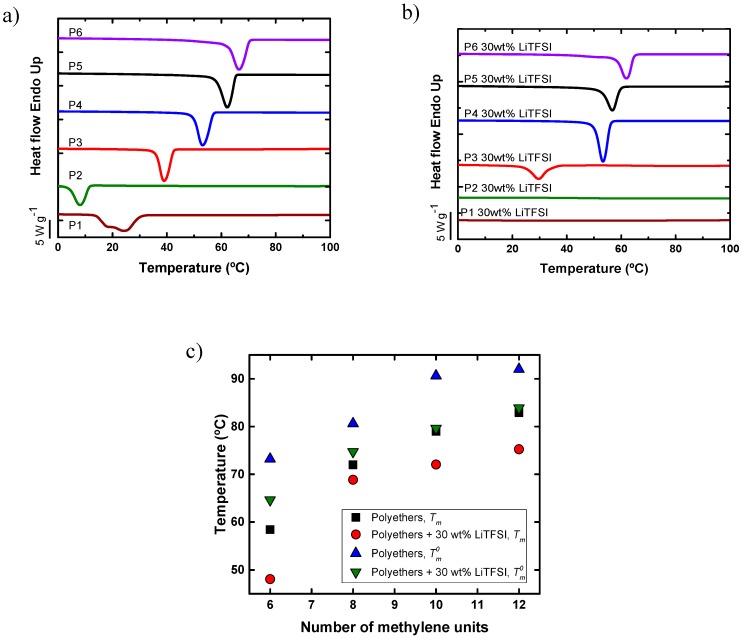
(**a**) Differential scanning calorimetry (DSC) cooling scans of neat aliphatic polyethers, (**b**) DSC cooling scans of polymer electrolytes composed of aliphatic polyethers including 30 wt % LiTFSI, (**c**) experimental melting peak values (*T_m_*) (determined during the second DSC heating runs) and equilibrium melting point values (*T*_*m*_^0^) determined by the Hoffman–Weeks extrapolation procedure (see Supplementary Information) after isothermal crystallization.

As a reference, Figure 2a shows the cooling scans for the neat aliphatic polyethers. The crystallization temperature generally increases between 10 and 65 °C as the number of methylene groups in the polyethers repeating units increases. The only exception being the P1 (PEO) sample; this sample also shows a bimodal distribution of crystallization temperatures whose origin is unknown as it would merit further studies outside the scope of the present work. As the number of methylene units increases, the polyethers start to behave in a similar way to polyethylene showing a higher *T_m_*, as the effect of the polar oxygen atom is progressively diluted by the aliphatic chain.

Additionally, Figure 2b shows the crystallization temperature of the polyether SPEs with 30 wt % LiTFSI. In all cases, homogenous SPEs were obtained, except for the SPE based on P6, as LiTFSI has poor solubility in P6. Figure 2b shows that P1 and P2 with 30% LiTFSI are completely amorphous materials. However, in the case of P3, P4, P5, and P6, the crystallization temperature decreases as compared with the neat polyethers, indicating that LiTFSI slows down the non-isothermal crystallization kinetics from the melt at 20 °C min^−1^.

Figure 2c represents the change in the melting temperature of the neat polyethers and the polyethers with 30 wt % of LiTFSI. As expected, the melting temperature increases with the number of methylene units and for any given polyether, it decreases with salt addition. The depression of the melting temperature with the addition of LiTFSI could be due to a dilution effect of the salt. This possibility is examined in detail below by varying the salt concentration in selected samples and applying the Flory–Huggins theory. Figure 2c also shows how the values of the equilibrium melting point change as a function of the number of methylene groups in the repeating units for synthesized polyethers. These values were obtained using the Hoffman–Weeks extrapolation for isothermally crystallized samples, as shown in the Supplementary Information. The *T*_*m*_^0^ values also follow the same trend with the number of methylene units, as the apparent or experimentally determined melting points, as expected. In Figure 2c, the data for P1 and P2 are not reported, as the Hoffman–Weeks extrapolation yielded unsatisfactory data, a fact that may be due to the lower molecular weight values for these samples.

Given the results obtained above, samples P3 and P5 were chosen in order to study in detail the effect of salt concentration on ionic conductivity and crystallization. P3 has the lowest *T_m_* and *T_c_* values while P5 has one of the highest *T_m_* and *T_c_* values, from the series of long chain polyethers synthesized in this work. We need to remark that the mixture of P6 and LiTFSI was not homogeneous, thus P6 was not chosen for further analysis.

**Figure 3:**
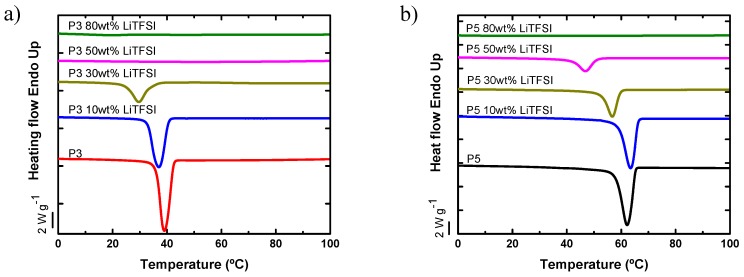
DSC cooling scans of neat P3 and P5 polyethers and their mixtures with different LiTFSI salt concentrations (solid polymer electrolytes: SPEs): (**a**) P3, (**b**) P5.

P3 and P5 were evaluated with different salt concentrations: 10, 30, 50, and 80 wt % LiTFSI. The general trends of crystallization temperature of either polyether were to decrease gradually with the addition of salt, as shown in Figure 3. This trend can be attributed to a dilution effect of the salt. In other words, the salt acts as a solvent that depresses both the crystallization and melting temperature of the polyether. In addition, P3 was completely amorphous with 50 wt % or more LiTFSI, whereas in the case of P5, the SPE-P% was rendered amorphous only when 80 wt % LiTFSI was added.

### 4.2. Ionic Conductivity of Aliphatic Polyethers in the Presence of LiTFSI

**Figure 4:**
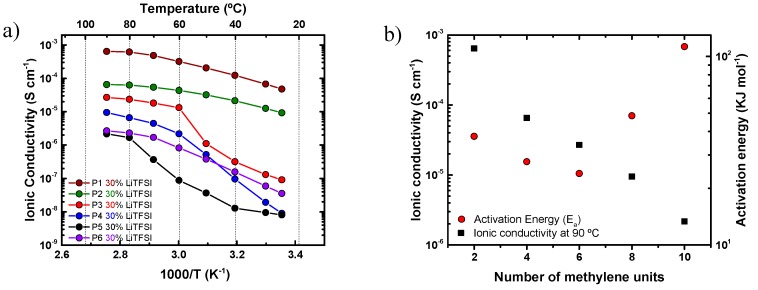
(**a**) Ionic conductivity of the polyethers with 30 wt % LiTFSI. (**b**) Ionic conductivity of the polyethers with 30 wt % LiTFSI at 90 °C and activation energy (*E_a_*) calculated in the molten state for the SPEs.

The ionic conductivity of these solid polymer electrolytes (SPEs) was studied by impedance spectroscopy. The ionic conductivity of polymer electrolytes with different salt contents was evaluated. First, the ionic conductivity of all polyethers with 30% LiTFSI was analyzed and the results are presented in Figure 4.

Figure 4a shows *a decrease in ionic conductivity* with the increase of the number of methylene groups along the repeating units of the polyethers employed. P1 and P2 provide the highest ionic mobility as they were amorphous. The amorphous nature of these materials can be deduced from their monotonic behavior in the Arrhenius representation plotted in Figure 4, and corroborated by DSC analysis, as shown in Figure 2b.

Figure 4a also shows how the ionic conductivity of P3, P4, P5, and P6 dramatically decreases as soon as these polyethers crystallize below 70 °C, as also shown in Figure 2b [24]. These data, where SPE-P1 provides the highest ionic conductivity, reveal that PEO is the best candidate, from the series of polyethers examined here, for hosting LiTFSI (ionic conductivity of 5·× 10^−4^ S cm^−1^ at 70 °C and 4.8 × 10^−5^ S cm^−1^ at room temperature). This high ionic conductivity can be explained by the favorable helical wrapping of Li ions on the polyether chain, when the ether oxygens are separated by exactly two carbon atoms [6]. This favorable coordination shows the highest ionic conductivity of the entire polyether family.

It should be noted that the behavior of SPE-P6 in Figure 4a was out of the general trend (i.e., the trend of decreasing conductivity as the number of methylene groups in the polyether repeating unit increases), due to its compromised solubility in the mixture of solvents and LiTFSI. Such poor solubility affects the homogeneity of the resulting SPE-P6. 

Figure 4b reports the ionic conductivity and the activation energies (*E_a_*) of the polyethers (except P6) at 90 °C (a temperature at which all samples are in the melt) in the linear region (only molten state data were employed); these values were calculated using the Arrhenius Equation [25]:

(1) $$\sigma =\sigma_{0}\exp(\frac{-E_{a}}{RT})$$

where *σ* is the ionic conductivity, *σ*_0_ is the pre- exponential factor, *E_a_* is the activation energy, *R* the universal gas constant, and *T* the absolute temperature.

For P1, P2, and P3, the activation energies obtained (*E_a_*) were of the same order of magnitude, but for P4 and P6 the activation energy significantly increased. In addition, a large decrease in conductivity with respect to the number of methylene groups in the repeating units of the polyethers was observed as expected. As a result, the ionic conductivity decreases with increasing *E_a_*. The activation energy values found in this work are similar to those reported in the literature [5].

**Figure 5:**
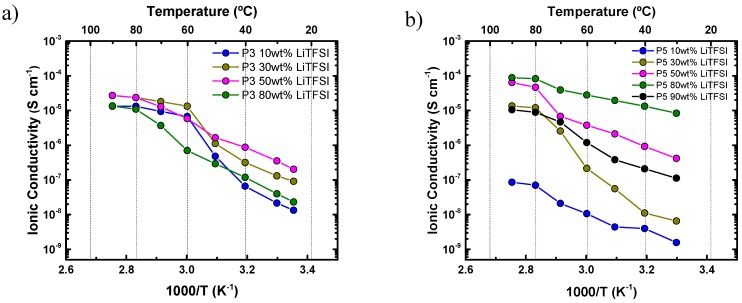
Ionic conductivity of SPE-P3 (**a**) and SPE-P5 (**b**) with different amounts of LiTFSI.

Among all SPEs, SPE-P3 and SPE-P5 were selected to study the effect of salt concentration (10, 30, 50, 80 wt % of LiTFSI) on the ionic conductivity, and the results are shown in Figure 5. The ionic conductivity of SPE-P3 increased with the increase of salt concentration from 10 to 50 wt %, whereas the crystallinity decreased, as shown in Figure 3a. The optimum ionic conductivity value at room temperature was 2.05·10^−5^ S cm^−1^, with 50 wt % LiTFSI. At higher salt concentrations the ionic conductivity of SPE-P3 decreased. In P5, the increase of ionic conductivity with the amount of salt is more evident; this value increased from 1.61·10^−9^ S cm^−1^ with 10 wt % LiTFSI to 8.8·10^−6^ S cm^−1^ with 80 wt % LiTFSI (at room temperature), and then with 90 wt % the conductivity dropped two orders of magnitude. In both cases, the conductivity was lower than with PEO with 30 wt % LiTFSI.

The degree of crystallinity (*X_C_*) of the polyether components was calculated for the two families of SPEs, using the following Equation:

(2) $$X_{C}=\frac{\Delta H_{m}}{f\;\Delta H_{m}^{0}}*100$$

where Δ*H*_*m*_^0^ is the equilibrium melting enthalpy for the 100% crystalline polyether, Δ*H_m_* is the experimental melting enthalpy of the sample, and *f* is the weight fraction of the polymer in the sample.

The values of Δ*H*_*m*_^0^ = 244.7 J g^−1^ for P3 and Δ*H*_*m*_^0^ = 258.3 J g^−1^ for P5 were employed. These values were obtained by the Flory–Huggins theory, where Δ*H*_*m*_^0^ is denoted as Δ*H_u_* (as explained below, in Section 4.4). Table 1 reports the crystallinity values obtained, and it was observed that the degree of crystallinity decreased as LiTFSI content increased, as was expected if the salt is considered a diluent for the polyethers. This crystallinity reduction is one of the reasons why SPEs exhibit a larger ionic conductivity as the content of LiTFSI increases.

### 4.3. Isothermal Crystallization of Aliphatic Polyethers in the Presence of LiTFSI

Isothermal crystallization experiments performed by DSC were useful to determine the overall crystallization kinetics of the polymeric samples employed here. These experiments were performed to study how the lithium salt concentration affects the overall crystallization kinetics of the different polyethers.

**Figure 6:**
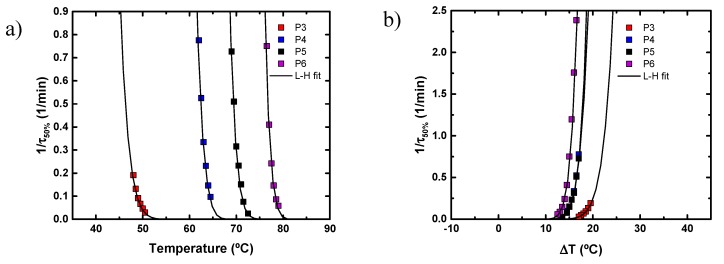
(**a**) Overall crystallization rate (expressed as the inverse of the half-crystallization time) versus isothermal crystallization temperature. (**b**) Overall crystallization rate (expressed as the inverse of the half-crystallization time) versus supercooling (Δ*T* = *T*_*m*_^0^ − *T_c_*). Symbols: experimental data. Solid lines show fits to the Lauritzen and Hoffman theory.

Figure 6a shows plots of the inverse of the half-crystallization time (1/*τ*_50%_) as a function of the crystallization temperature (*T_c_*) of the neat polyethers. The inverse of the half-crystallization time (1/*τ*_50%_) is an experimentally determined value that is directly proportional to the overall crystallization rate [21,26]. The crystallization temperature values where the kinetics were able to be measured decreased as the number of methylene units decreased in the polyether repeating unit. If the supercooling is calculated as Δ*T* = *T*_*m*_^0^ − *T_m_*, employing the equilibrium melting temperature values determined by the Hoffman–Weeks extrapolation, the results of which are shown in the Supplementary Information and in Figure 2, the overall crystallization kinetics can be represented as a function of supercooling. Figure 6b shows how the overall crystallization kinetics plots for the different polyethers are now much closer together (using the same relative temperature range), since the supercooling normalizes the plot with respect to thermodynamic effects. The supercooling required for crystallization decreases as the number of methylene units in the polyether increases, as shown in Figure 6b. This result is consistent with the non-isothermal crystallization data reported in Figure 3.

**Figure 7:**
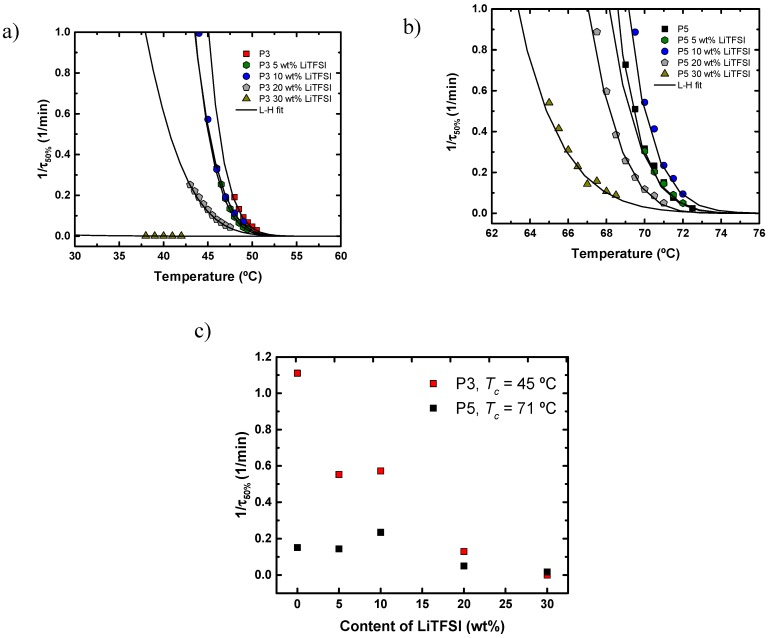
(**a**) Overall crystallization rate (expressed as the inverse of the half-crystallization time) versus isothermal crystallization temperature for P3 and SPEs-P3. (**b**) Overall crystallization rate (expressed as the inverse of the half-crystallization time) versus isothermal crystallization temperature for P5 and SPEs-P5. Symbols: experimental data. Solid lines show fittings to the Lauritzen and Hoffman theory. (**c**) Overall crystallization rate (expressed as the inverse of the half-crystallization time) versus the content of LiTFSI at constant crystallization temperatures (notice that T_c_ values are different for each series), whose values are indicated in the legend.

The overall crystallization rate of P3 and P5 with different amounts of LiTFSI (5–30 wt %) was studied and the results are presented in Figure 7. In the case of the SPE-P3 samples, the crystallization rate decreased with salt content, a result that is explained by the dilution effect caused by LiTFSI. Similar results were obtained for SPE-P5 samples, except for the sample with 10 wt % LiTFSI, which exhibited a larger value than expected. Apart from this particular sample, all the rest behaved as expected and the results indicate that the overall crystallization kinetics of these polyethers was substantially depressed by the incorporation of LiTFSI. The best way to visualize this change in crystallization rate is to plot the overall crystallization rate at a constant temperature (45 °C in the case of P3 and 71 °C for P5) as a function of LiTFSI content, as shown in Figure 7c. The results clearly show that the overall crystallization rate of polyethers P3 and P5 generally decrease with increasing LiTFSI content.

### 4.4. Diluent Effect of LiTFSI

LiTFSI may act as a solvent that depresses the melting temperature of polyethers [27]. In order to demonstrate if LiTFSI behaves like a low molecular weight diluent, we have employed the Flory–Huggins theory for polymer–diluent mixtures [28,29]. The fundamental Equation can be written as:

(3) $$\frac{\frac{1}{T_{m}}-\frac{1}{T_{m}^{0}}}{\upsilon_{1}}=\frac{R}{\Delta H_{u}}\frac{V_{u}}{V_{1}}\left(1-\frac{{\mathrm{BV}}_{1}}{R}\frac{\upsilon_{1}}{T_{m}}\right)$$

where Δ*H_u_* is the melting enthalpy per mole of repeating unit, V_u_ and V_1_ are the molar volumes of the polymer repeating unit and the diluent, respectively, υ_1_ is the volume fraction of the diluent, B is the interaction energy density character of the polymer-diluent pair, *T*_m_ is the apparent melting temperature (taken from the DSC second heating run), and *T*_m_^0^ is the equilibrium melting temperature (determined by the Hoffman–Weeks extrapolation method), as shown in the Supplementary Information and Figure 2. All temperatures are expressed in Kelvin degrees and *R* is the gas constant.

**Figure 8:**
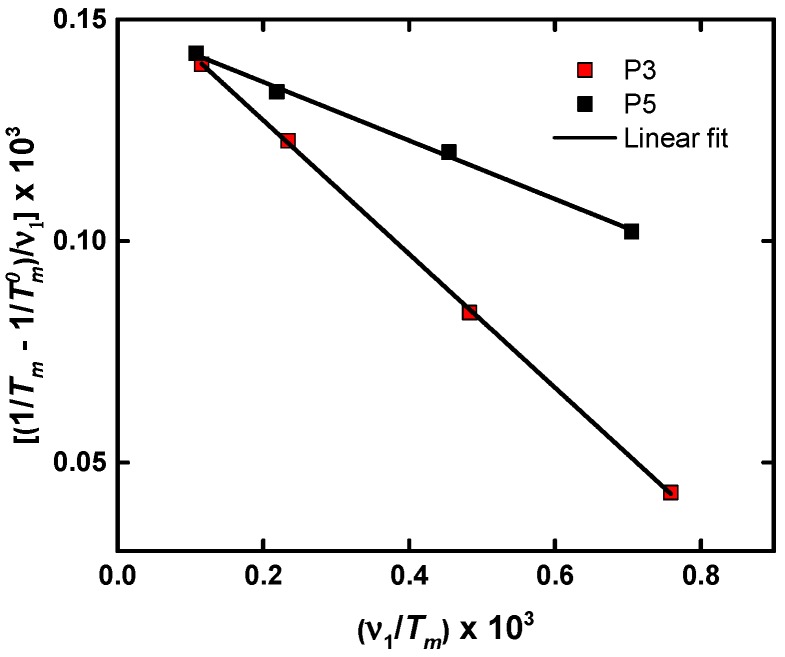
Graph of [(1/*T*_m_ − 1/*T*_m_^0^)/υ_1_] × 10^3^ as a function of (υ_1_/*T*_m_) × 10^3^.

Figure 8 shows that the plot of [(1/*T*_m_ − 1/*T*_m_^0^)/υ_1_] × 10^3^ as a function of (υ_1_/*T*_m_) × 10^3^ is a straight line. This linear relationship indicates that the Flory–Huggins theory was obeyed for LiTFSI and polyether (P3-P5) mixtures, or in other words, that LiTFSI acts as diluent for the employed polyethers.

From the intercept of the straight line it is possible to obtain the value of ΔH_u_, whereas from the slope, the value of B is determined. It has been demonstrated that the value of ΔH_u_ was a property of the crystallizing chain repeating unit and did not depend on the nature of the diluent. It is therefore a fundamental thermodynamic property of the polyether crystal that is directly related to its chain structure. ΔH_u_ is therefore the enthalpy of melting of a 100% crystalline material expressed as the heat of fusion per repeating unit [29]. The values obtained from Figure 8 for P3 are ΔH_u_ = 24,741 J mol^−1^ and B = 3.7 J cm^−3^, and for P5, ΔH_u_ = 40,300 J mol^−1^ and B = 48 J cm^−3^. The value of ΔH_u_ for P3 is similar to that reported in the literature [29], i.e., 23,640 J mol^−1^.

**Figure 9:**
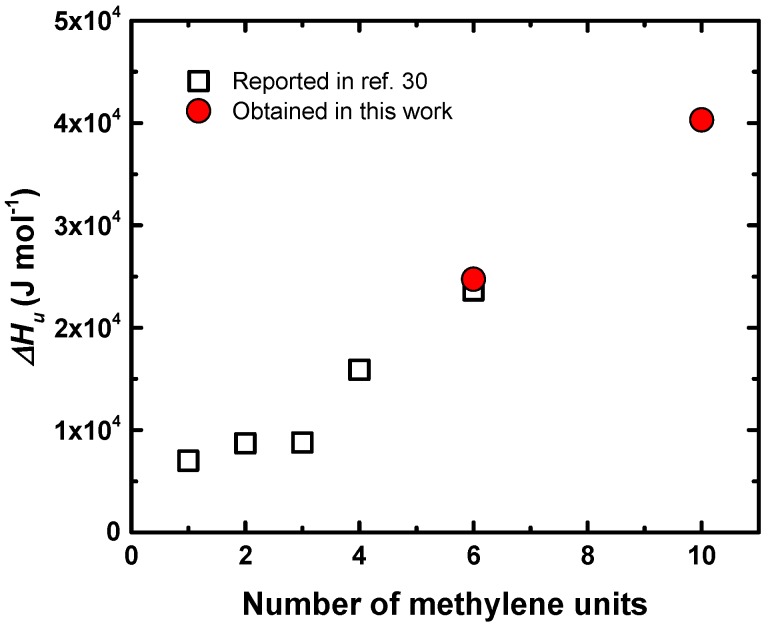
ΔH_u_ values of polyethers as a function of the number of methylene units in the repeating unit. The black points are values reported in the literature [29] and the red points are the values obtained in this work.

In the case of P5, the value of the equilibrium melting temperature and equilibrium melting enthalpy were reported in this work for the first time, as far as the authors are aware. Figure 9 shows the data of some polyethers reported in the literature [29] and the experimental values obtained here by the application of the Flory–Huggins theory to our data. It can be seen that the values of ΔH_u_ increased with the number of –CH_2_– and in the case of P3 (six methylene units), our value was very similar to that reported in the literature, as already mentioned. In addition, the value for P5 (10 methylene units) followed the expected trend.

The value of B is related to the polymer–diluent interaction and therefore it depends on the chemical structure of the diluent component. The different slopes that are observed in Figure 8 reflect differences in the Flory–Huggins interaction parameters for the mixtures. The Flory–Huggins polymer–diluent interaction parameter (*χ*_1_) can be expressed as [28]:

*χ*_1_ = *κ*_1_ − *ψ*_1_ + 1/2 (4)

where *κ*_1_ and *ψ*_1_ are enthalpic and entropic parameters related to the partial molar enthalpy ΔH_1_ = RT *κ*_1_υ_2_^2^ and the partial molar entropy ΔS_1_ = RT *ψ*_1_ υ_2_^2^. The enthalpic term can also be represented as:

*κ*_1_ = *B*V_1_/RT. (5)

As can be seen from the above equations, B is directly proportional to the enthalpic contribution (*κ*_1_) to the Flory–Huggins parameter *χ*_1_. The lower the value of *χ*_1_, the higher the thermodynamic interaction between the polymer and the diluent. The B values obtained from Figure 8 are B = 3.7 J cm^−3^ for P3, and B = 48 J cm^−3^ for P5. Therefore, LiTFSI was a better solvent for P3 than for P5, an expected result based on the chemical structure of P3 and P5, as the polarity degree within the polyether molecules decreased as the number of methylene units increased along the repeating unit. From the application of the Flory–Huggins theory, we can extrapolate the results to conclude that small values of B are necessary to increase the ionic conductivity.

## 5. Conclusions

In this paper the impact of the chemical structure of aliphatic polyethers and salt concentration on crystallization rate, crystallization temperature, and ionic conductivity has been investigated. As a general observation, the LiTFSI salt acts as a diluent for all the aliphatic polyethers, reducing the crystallization rate and crystallization temperature. The ionic conductivities of the SPEs were obtained in the order of 10^−8^–10^−4^ S cm^−1^ at 70 °C. A higher number of methylene units in the polyether repeating unit caused a decrease in the ionic conductivity of the SPEs in the following order PEO (P1) > P(THF) (P2) > poly(oxyhexamethylene) (P3) > poly(oxyoctomethylene) (P4) > poly(oxydecamethylene) (P5) > poly(oxydodecamethylene) (P6) with 30 wt % of LiTFSI. The reason for this behavior is probably due to the decreasing solvation of lithium atoms in the same order. Additionally, as salt concentration increased, both crystallization temperature and melting enthalpies of the SPE-P3 and SPE-P5 were found to decrease. By applying the Flory–Huggins theory, we demonstrated that LiTFSI acts as a thermodynamic diluent for the polyethers examined. The interaction energy parameter (*B*) was calculated for SPEs prepared with P3 and P5. We showed that the value of *B* must be small to obtain high ionic conductivity electrolytes. In the case of the poly(oxydecamethylene), the value of the equilibrium melting temperature and equilibrium melting enthalpy were reported in this work for the first time, as far as the authors are aware.

## Figures and Tables

**Table 1:** Δ*H_m_* and degree of crystallinity of SPE-P3 and SPE-P5 with different amounts of LiTFSI.

Sample	Δ*H_m_* (J g^−1^)	Crystallinity (%)
**P1**	149	69
**P2**	85	36
**P3**	127	51
**P3 10 wt % LiTFSI**	99	45
**P3 30 wt % LiTFSI**	49	29
**P3 50 wt % LiTFSI**	0	0
**P4**	145	56
**P5**	135	52
**P5 10 wt % LiTFSI**	119	51
**P5 30 wt % LiTFSI**	80	44
**P5 50 wt % LiTFSI**	47	36
**P5 80 wt % LiTFSI**	0	0
**P6**	142	53

## Supplementary Materials

The following are available online at https://www.mdpi.com/2073-4360/11/3/452/s1.
