# Supplementary material for: Effect of Chemical Structure and Salt Concentration on the Crystallization and Ionic Conductivity of Aliphatic Polyethers
Source: Polymers (Basel). 2019 Mar 9;11(3):452. doi: 10.3390/polym11030452 (PMC6473696; doi:10.3390/polym11030452)
Supplement: Supplementary file 1 [file polymers-11-00452-s001.pdf]

Supporting Information

Effect of chemical structure and salt concentration on the crystallization and ionic conductivity of aliphatic polyethers

By

Jorge L. Olmedo-Martínez[a], Leire Meabe[a], Andere Basterretxea[a], David Mecerreyes[a,b], Alejandro J. Müller[a,b*]

[a] POLYMAT and Polymer Science and Technology Department, Faculty of Chemistry, University of the Basque Country UPV/EHU, Paseo Manuel de Lardizabal 3, 20018 Donostia-San Sebastián, Spain

[b] IKERBASQUE, Basque Foundation for Science, 48011 Bilbao, Spain

***Corresponding author**

Solutions of polyethers in chloroform

Figure S1 show that P1 to P5 are completely soluble in chloroform. In the case of P6 the material is not completely soluble at room temperature (notice the cloudiness of the solution), for this reason the ionic conductivity has an out-of-trend behavior.

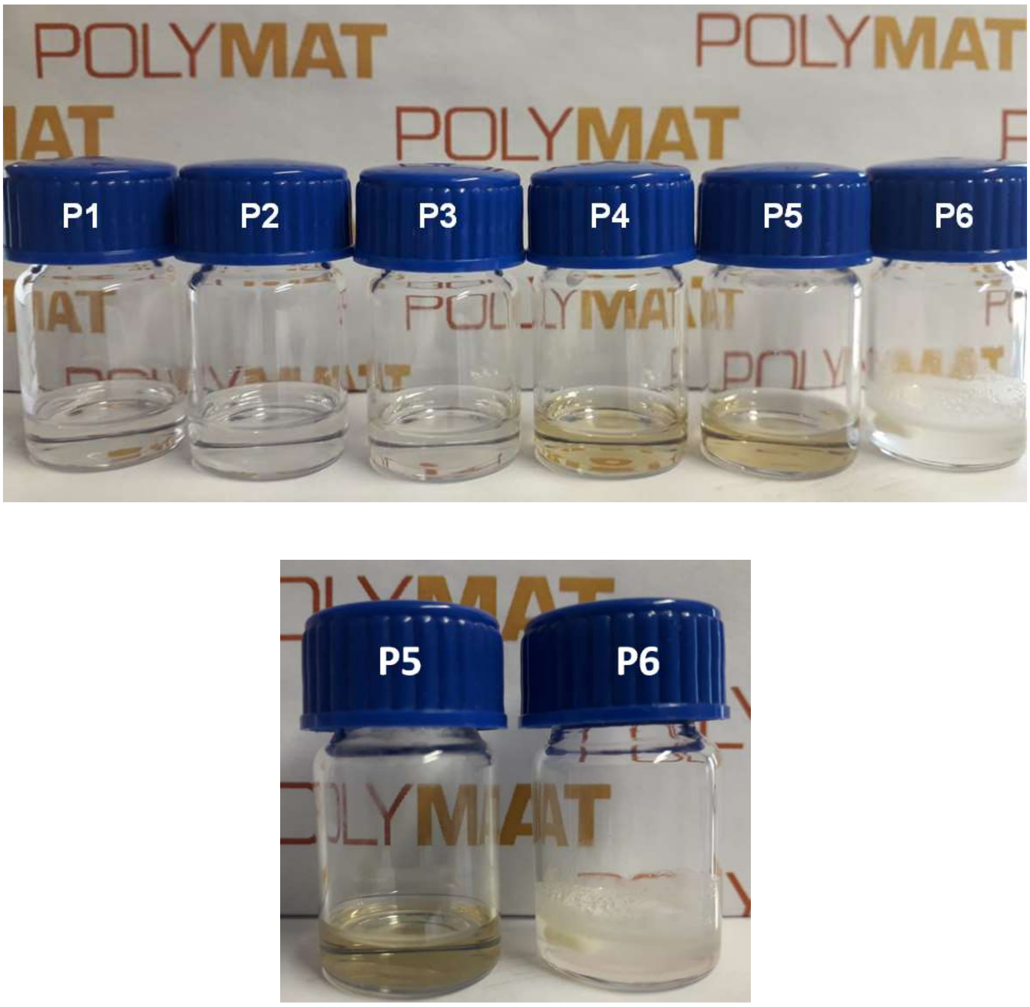

Figure S1. Photos of the solutions of the polyethers in chloroform.

Electrochemical impedance spectroscopy (EIS)

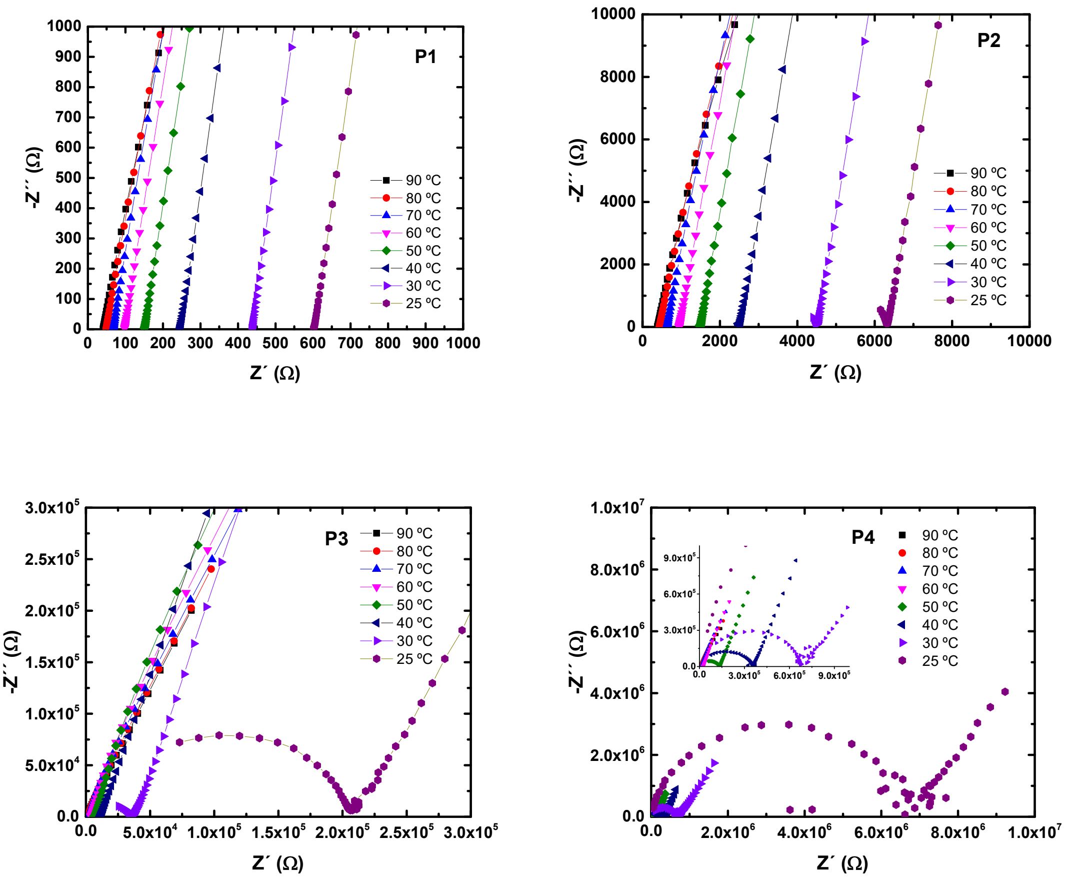
P1
P2
P3
P4
-Z´´ (Ω)
Z´ (Ω)
90 ºC
80 ºC
70 ºC
60 ºC
50 ºC
40 ºC
30 ºC
25 ºC

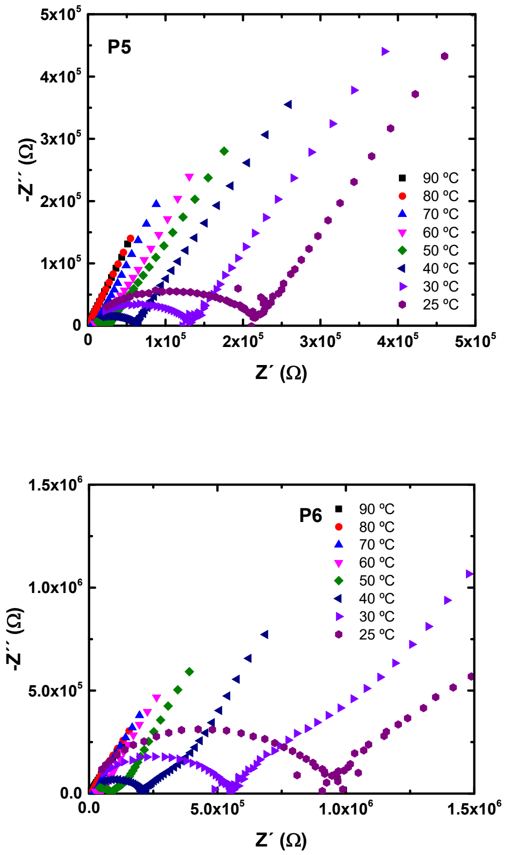

Figure S2. Nyquist plots of polyethers with 30 wt% LiTFSI.

Hoffman- Weeks extrapolation

To perform the Hoffman-Weeks extrapolation, the samples were heated after isothermal crystallization from their T_c values until melting and the peak melting temperature (T_m) were recorded. As an example, the Hoffman-Weeks plots for P3, P4, P5 and P6 are shown in the Figure S3. The straight line of the observed melting temperature against T_c intersects with the equilibrium line (red line T_m = T_c) [1,2,3]. From the extrapolations, we estimated the T_m^0 values for all the samples.

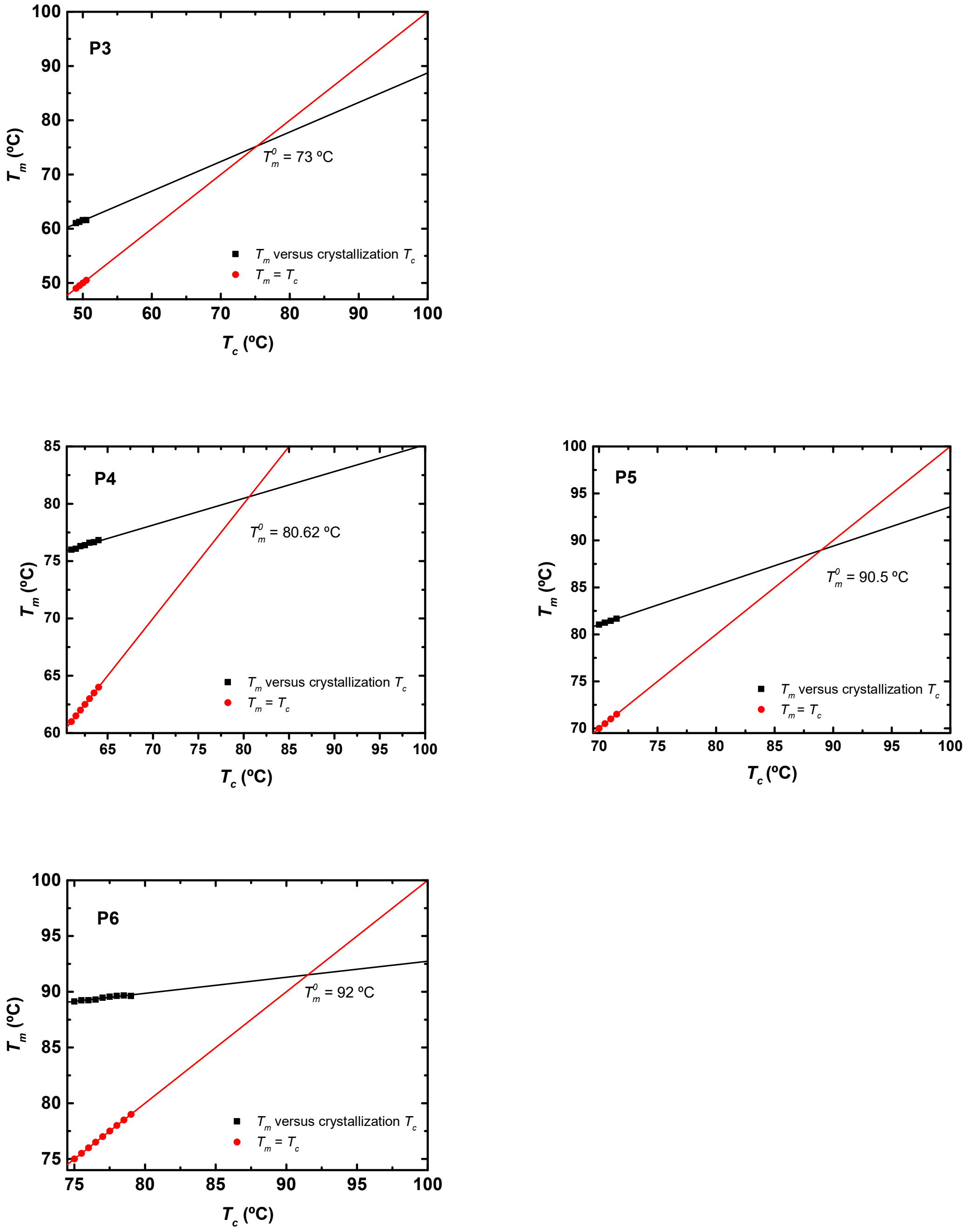

Figure S3. Hoffman- Weeks plots of neat polyethers

Flory Huggins Theory

Table S1 shows the parameters used and the calculated values for SPE-P3 and SPE-5, after applying the Flory-Huggins theory.

Table S1. Calculated data for (υ_1/T_m) x 10^3 versus [($1/Tm - 1/T_m^0$)/υ_1] x10^3

Sample	υ_1	T_m (K)	T_m^0 (K)	($\upsilon 1/T_m$) x 10^3	[($1/Tm - 1/T_m^0$)/$\upsilon 1$] x10^3
P3 5 wt% LiTFSI	0.038	329.78	330.36	0.1154	0.1398
P3 10 wt% LiTFSI	0.077	329.61	330.64	0.2339	0.1225
P3 20 wt% LiTFSI	0.158	327.59	329.02	0.4830	0.0838
P3 30 wt% LiTFSI	0.248	321.22	322.31	0.7587	0.0432
P5 5 wt% LiTFSI	0.038	353.9	354.6	0.1075	0.1423
P5 10 wt% LiTFSI	0.077	353.16	354.2	0.2183	0.1336
P5 20 wt% LiTFSI	0.158	348.28	350.6	0.4843	0.1200
P5 30 wt% LiTFSI	0.248	345.73	348.73	0.7049	0.1021

Where T_m is the apparent melting point, T_m^0 is the equilibrium melting temperature, υ_1 is the volume fraction of LiTFSI, ΔH_u is the melting enthaply per mole of repeating unit [3].

References

[1] A.T. Lorenzo, M.L. Arnal, J. Albuerne, A.J. Müller, DSC isothermal polymer crystallization kinetics measurements and the use of the Avrami equation to fit the data: Guidelines to avoid common problems, Polym. Test. 26 (2007) 222–231. doi:10.1016/j.polymertesting.2006.10.005.

[2] A.T. Müller, Alejandro J., Michell, R. M., Lorenzo, Isothermal Crystallization Kinetics of Polymers, in: Q. Guo (Ed.), Polym. Morphol. Princ. Charact. Process., John Wiley & Sons, 2016: pp. 181–203. doi:https://doi.org/10.1002/9781118892756.ch11.

[3] L. Mandelkern, Crystallization of Polymers, 2nd ed. Vo, Cambridge University Press, Cambridge, United Kingdom, 2002.
